# Effectiveness of Web Applications on Improving Nutritional Status of Patients with Colorectal Cancer

**DOI:** 10.3390/nu16030408

**Published:** 2024-01-30

**Authors:** Pornpimon Nunthanawanich, Sanit Wichansawakun, Cholrit Luangjinda, Chatrapa Hudthagosol

**Affiliations:** 1 Doctor of Public Health (International Program), Faculty of Public Health, Mahidol University, Bangkok 10400, Thailand; 2 Division of Clinical Nutrition, Department of Internal Medicine, Faculty of Medicine, Thammasat University, Pathum Thani 12120, Thailand; wichansawakun@gmail.com; 3 Research and Development Office, Thailand Institute of Occupational Safety and Health, Ministry of Labor, Bangkok 10170, Thailand; 4 Department of Nutrition, Faculty of Public Health, Mahidol University, Bangkok 10400, Thailand

**Keywords:** mHealth, web application, nutrition status, malnutrition, colorectal cancer

## Abstract

The most common cancer in Thailand is colorectal cancer (CRC). A lack of knowledge and misleading information from social media have contributed to cancer deaths from malnutrition. A web application is a tool that provides easy access to scientific nutritional information via an online platform. In this study, our goal was to compare the nutritional status of CRC patients using different nutrition-based educational tools with nutrition counseling, namely the Nutrition Educational Prototype based on Smartphone Web Applications (NEPSA) and standard hospital leaflets. Anthropometric and biochemical analyses and a dietary assessment, especially calories and protein, were measured during three visits. This study finally included 28 CRC patients who were undergoing chemotherapy and malnutrition with a body mass index (BMI) of <20 kg/m^2^. Thirteen participants received NEPSA while the remaining fifteen participants received a standard hospital leaflet. The results showed that NEPSAs improved nutritional outcomes by encouraging weight gain, increasing BMI, hemoglobin, hematocrit, and albumin levels, and consuming more calories and protein. NEPSA should be implemented to enhance the nutrition outcomes from anthropometric, biochemical, and dietary perspectives from nutrition advice among CRC patients. There could be positive impacts at the national level regarding equal accessibility to Thailand’s nutrition information.

## 1. Introduction

Colorectal cancer is the most common type of cancer in both males and females worldwide, including Thailand [1,2]. The number of cases increased from 3416 to 6068 between 2014 and 2018, a rise of approximately 45.2% over five years in Thailand [1]. Chemotherapy and radiotherapy can cause gastrointestinal problems such as loss of appetite, taste perception change, nausea, vomiting, constipation, and diarrhea [3]. These symptoms can lead to poor dietary intake-induced malnutrition [3,4]. Various factors including cytokines, metabolic changes, and emotional distress [3,4,5,6,7], contribute to malnutrition or cancer cachexia, a major cause of death in CRC [5]. Lack of nutritional knowledge contributes to malnutrition and complicates treatment, leading to longer hospital stays, contracting infections more easily, or complications [4,8,9]. The doctor can refer malnourished patients to a dietitian, but it is often too late; patients will likely have a poor quality of life and experience difficulty following nutritional recommendations [10]. Other studies have found that nutrition knowledge is linked to healthy eating practices [9,10,11,12,13].

Health education is a cost-effective way to improve malnutrition in cancer patients [11,12,13,14,15,16]. It increases dietary intake and helps with self-confidence and food choices [15]. Nowadays, people can easily find unclear nutrition information on social media. According to a technology use survey in Thailand, nearly 40% of smartphone owners have searched for health information [17]. The impact of information impact on society is crucial for cancer groups because it influences the risk of malnutrition and recurrence, as previously mentioned. Thailand’s public health system faces a new challenge: combining nutritional education with smartphone apps. Previous studies have shown that smartphone applications are helpful for cancer patients [18,19,20,21,22]. They are easy to access and have convenient features. Thus, an alternative and interesting nutrition-based educational tool in this digital era can provide reliable nutritional information for cancer patients undergoing chemotherapy, resulting in less toxic and cost-effective treatments afterward.

Cancer patients face a knowledge gap due to a lack of dietetic services and limited time. The patient also faces certain aforementioned obstacles during chemotherapy. The internet cannot always provide reliable information based on nutrition guidelines [23]. Therefore, combining nutrition guidelines using science and technology is a tangible solution. At the national level, this should improve nutrition and democratize nutritional information.

## 2. Materials and Methods

### 2.1. Study Design

A quasi-experimental study (pre-test and post-test designs) with a non-equivalent control group was conducted from August 2021 to October 2022 at the Faculty of Medicine, Thammasat University Hospital, Pathum Thani, Thailand. A convenient sample was calculated from a similar research design using a *t*-test [24]. Match-group comparison was designed in this study. At least 11 participants from each group were settled, including 20% of those who did not participate.

This study recruited 453 CRC patients in that period from the oncology unit. Only 32 participants passed the eligibility assessment for inclusion criteria: those who were diagnosed with CRC between 30 and 75 years old with a BMI of <20 kg/m^2^ who planned to receive intravenous chemotherapy, were able to eat orally, can communicate in Thai, and had a smartphone or tablet with screen time of at least 1 h per day. We excluded those with complications such as gut obstruction, brain dysfunction, gastrointestinal dysfunction, or metastasis. Additionally, we excluded those who experienced critical events. Two participants were excluded from the initial screening because their families disagreed based on negative experiences in previous studies.

**Figure 1:**
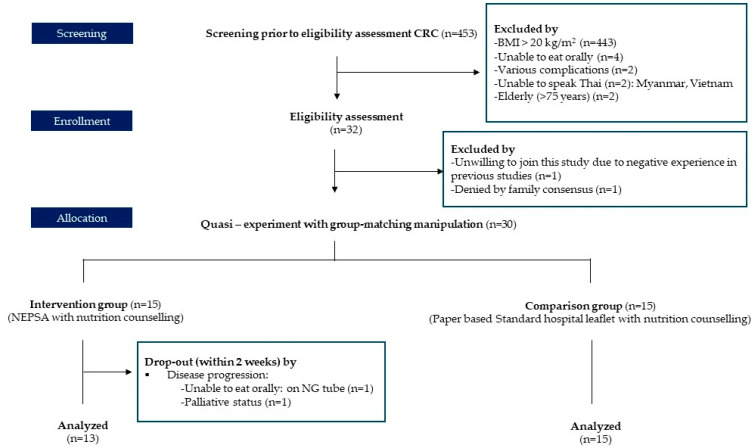
Intervention enrollment.

In total, 30 participants were included in this study. Two participants left the study due to disease progression. They were unable to eat orally and had to rely on a nasogastric tube (NG). Additionally, they had a palliative care plan at the time of their first visit. Thus, we conducted our statistical analysis based on 28 participants, as shown in Figure 1. The intervention group was supplied with a Nutrition Educational Prototype based on a Smartphone Web Application (NEPSA) as a tool along with nutrition counseling, which included six modules:

- Nutritional news: scientific information combatting misleading information shown on social media.
- Self-weekly body weight recorder: motivates patients to monitor their body weight with a self-weekly body weight recorder.
- Food group selection: proper nutritional information on how to make appropriate food choices during chemotherapy based on food groups, e.g., carbohydrates, protein, fat, fruit, and vegetables.
- Fruit and vegetable washing techniques: three easy washing methods followed by public health concerns that can be practiced at the household level.
- Therapeutic recipes with diet tricks for reducing chemotherapy side effects: 50 therapeutic recipes with cooking videos. The menu function offers cooking tips for decreasing chemotherapy side effects, including recipes for reducing nausea, preventing diarrhea, stimulating appetite, and other related issues.
- Medical food selection: informs on commercial oral nutritional supplements (ONS) available in Thailand and lists the beneficial properties of each formula with automatic scoop calculation features.

Additionally, the comparison group received nutrition counseling using standard hospital leaflet as a tool with six modules, as described below:

- Food group selection: proper nutritional information on making appropriate food choices during chemotherapy based on food groups, e.g., carbohydrates, protein, fat, fruit, vegetables, and water intake.
- Meal plan examples with a table of calorie requirements: 1500 kcal/1800 kcal and 2000 kcal with specific portion sizes of food groups: starch/protein (meat)/milk or dairy product/oil/fruit and vegetables.
- ONS: suggests medical formulas that provide immunonutrients to cancer patients who do not achieve nutritional goals.
- Eating guidelines when suffering from chemotherapy side effects: information on reducing treatment side effects, such as poor appetite, nausea and vomiting, mucositis, chewing and swallowing difficulty, constipation, diarrhea, neutropenia, and changes in taste.
- Food avoidance in cancer patients.
- Diet tricks: nutritional tricks to reach nutritional goals with small, frequent meals containing calorie-dense foods and a protein-based diet.

Both the intervention and comparison groups received personal nutrition counselling from an experienced registered dietitian (RD) at their first visit by followed ESPEN guidelines for cancer patients [23]:

- A calorie-dense and regular protein diet with small, multiple meals were distributed to participants individually.
- Carbohydrate suggestions followed by gastrointestinal symptoms and underlying diseases.
- Lean protein from breast chicken, fish, white egg, tofu, and skinless meat were suggested to be cooked well-done in proper portion sizes.
- Mainly monounsaturated fatty acid (MUFA) oil was suggested as a trick for adding more calories to limited portions.
- Immunonutrient supplementation (omega-3, arginine, and nucleotides) during the day was suggested (as a snack or after resistance exercise) to provide adequate calories and protein.
- In terms of physical activity, increasing resistance exercise in addition to aerobic exercise was suggested to maintain muscle mass and prevent muscle atrophy.

### 2.2. Data Collection

This study was conducted in three visits according to the oncologist’s chemotherapy appointment plans. Oncologists’ treatment guidelines were modified to reduce hospital visits during the COVID-19 situation. Two chemotherapy regimens, FOLFOX and CAPOX, which have 14- and 21-day cycles, respectively, were suggested depending on oncologists’ recommendations. Bioelectric impedance analysis (BIA) with TANITA–330 was used for anthropometric assessment. The machine was calibrated before every intervention visit to ensure reliable numeric values. Hemoglobin, hematocrit, total protein, globulin, albumin, and ESR were analyzed. Medical technicians collected blood once as per the oncologist’s instructions to avoid any invasive procedures on the participants. Dietary assessment using 24-h recall was conducted following the ethics committee’s suggestion of reducing the burden on participants. An experienced registered dietitian conducted the anthropometric and dietary assessments.

### 2.3. Ethical Approval

This study was conducted during the COVID-19 pandemic under Thammasat University Hospital regulations, which are in accordance with the Declaration of Helsinki, and approved by the Ethical Review Committee for Human Research of the Faculty of Public Health, Mahidol University (protocol no. 111/2020 COA No. MUPH 2020–148) on 23 November 2020. Thammasat University’s Human Research Ethics Committee at the Faculty of Medicine approved the research protocol MTU-EC-OO-4-284/63 on 27 May 2021. Written informed consent was obtained from all study participants.

### 2.4. Data Analyses

SPSS version 18 (SPSS (Thailand) Co., Ltd., Bangkok, Thailand) Mahidol University licensed for Windows was used for statistical analyses. A Shapiro–Wilk test assessed the normal distribution of all indicators. Descriptive data were presented as the mean ± SD and percentage. A chi-square test was used to compare demographic data between the experimental and comparison groups. Paired and independent *t*-tests were used to evaluate the differences in normally distributed variables within and between the groups, respectively. The Mann–Whitney U and signed rank tests were used to evaluate the differences in distribution within and between the groups, respectively. Repeated-measures ANOVA using Bonferroni’s test was used to evaluate differences in normally distributed variables within the group. Friedman’s and Wallis’ analyses of variance were used within and between the groups, respectively, during three intervention visits. Statistical significance was considered when *p* < 0.05. Friedman’s analysis of variance was considered statistically significant when *p* < 0.017.

## 3. Results

### 3.1. Baseline Charateristics

In total, 28 participants were analyzed in this study. The groups did not significantly differ in characteristics, including gender, age, marital status, educational level, monthly income, residence, cancer staging, and chemotherapy formula. Most of participants in our study were CRC stage 4, namely 84.62% in intervention group and 80% in the comparison group. The mean age of the intervention group was 63.77 ± 2.26 while that of comparison group was 61.07 ± 2.12. Both groups contained 100% non-alcohol users and non-smokers. Table 1 shows a comparison between the study groups.

### 3.2. Anthropometric Assessment

The baseline characteristics of anthropometric parameters were not significantly different between groups. During the second intervention visit (Visit2), the intervention group showed significant improvements in all measurements, whereas the comparison group did not present any significant changes. After implementation, the intervention group’s body weight, BMI, fat mass, and muscle mass increased significantly compared to baseline. However, the comparison group’s anthropometric parameters did not change significantly, with only body weight and BMI significantly improved compared to baseline at the end of the intervention, as shown in Table 2.

### 3.3. Biochemical Assessment

Biochemical parameters were not significantly different from baseline between groups. During implementation (Visit2 of the intervention), only hemoglobin, hematocrit, and albumin levels in the intervention group had significantly improved compared to baseline. At the end of implementation, hemoglobin, hematocrit, total protein, albumin levels, and ESR in the intervention group had significantly improved compared to baseline. No biochemical parameters significantly changed in the comparison group. Only total protein, globulin, and albumin levels were significantly changed between groups after implementation, as shown in Table 3.

### 3.4. Dietary Assessment

The INMUCAL 4.0 program (Institution of Nutrition, Mahidol University licensed) is a standard Thai program developed by the Institution of Nutrition, Mahidol University, Thailand, for calculating nutritional values. It is generally used to analyze the nutritional value of macronutrients, micronutrient, and provide other Thai traditional food nutrition facts. Important nutritional values in clinical cancer practice include total calories and protein intake.

Calorie and protein intakes were not significantly different from baseline between groups. Calorie and protein intakes had increased significantly by visiting dependents within the intervention group. Only protein intake was significantly elevated at the end of implementation in the comparison group. However, no significant differences were detected at the end of implementation when comparing the calorie intake between the intervention and comparison groups.

In terms of protein intake, the intervention group was significantly higher than the comparison group. It presented itself as significantly higher by visiting dependents. Total protein intake in the intervention group was elevated at 32.24 ± 4.68 g, whereas the comparison group increased by only 14.42 ± 4.09 g. A considerable change in protein intake, of up to 74.35%, was detected at the end of implementation in the intervention group, as shown in Table 4.

## 4. Discussion

From the anthropometric results, NEPSA could improve body weight and BMI more efficiently than the comparison group within three visits of implementation. This finding demonstrates the beneficial health outcome of providing appropriate nutritional transformation via the mHealth platform to fragile populations who must receive proper nutritional consultation to reduce the risk of cancer cachexia [25,26,27]. Cancer cachexia, known to reduce skeletal muscle mass, always occurs in malnourished cancer patients because cancer-induced inflammation is associated with weight loss. Gaining muscle mass is difficult for cancer patients because the synthesis of skeletal muscle protein is suppressed by inflammation [28]. Surprisingly, our study showed an increase in muscle mass only in the intervention group when comparing the second and third visits to baseline, as shown in Table 2. Decreasing inflammation (ESR) levels in the body, as shown to occur in Table 3, may help improve protein synthesis in muscle mass [29]. In addition, a sufficient intake of calories and protein, as shown in Table 4, can substantially impact total body weight. Physical activity recommendations following ESPEN guidelines, also mentioned at the baseline visit, include daily walking, gradually increasing resistance exercise, and supplementing immunonutrition with ONS, which may be another method of supporting muscle mass gain [23]. Reduced hemoglobin and hematocrit were generally observed in CAPOX and FOLFOX regimens due to cancer-induced inflammation [30,31,32,33]. According to previous studies, improving hemoglobin and hematocrit levels may positively impact quality of life, clinical status, and survival rates [34], and we also point out similar trends in our study. The participants in the NEPSA group had elevated hemoglobin and hematocrit level as shown in Table 3. Additionally, albumin levels are a crucial biochemical factor in nutrition [35]. Insufficient nutritional support, especially calorie and protein intake, is directly associated with hypoalbuminemia, which is an indicator of malnutrition [36,37]. Our study results showed that raising albumin levels was also significantly associated with reduced ESR. In the intervention group at the end of the study, following a similar trend as the previous observation when albumin was higher, inflammation was lessened after the patients received adequate nutritional support from calories and protein [38,39].

Regarding dietary assessment, calorie intake was raised by 35.17% compared to the first visit in the NEPSA group. By contrast, a 23.62% increase in calorie intake was observed in the comparison group after three intervention visits. These findings reflected a change in eating behaviors caused by NEPSA’s six nutrition modules, which provide proper nutritional information related to 50 therapeutic recipes, diet tips to relieve chemotherapy side effects, cooking preparation, fruit and vegetable washing techniques, and foods for lifestyle modification during chemotherapy. The previous BENECA mHealth app was an innovative tool for determining reliable calorie balance and promoting practical lifestyle changes, which the authors of our study also approved [40]. Experts used a smartphone application to facilitate and deliver nutrition and dietary counseling for Malaysian cancer survivors, as well as pain management for adolescents with cancer [41]. By making nutritional information more accessible, this platform can promote health equality, thereby improving cancer patients’ quality of life [40,41,42,43,44,45,46]. In theory, nutrition literacy refers to the ability to make informed decisions about food and its impact on health through knowledge, skill, and attitude [47]. Promoting nutrition literacy among cancer patients is a public health-based approach to reducing malnutrition and improving anthropometric and biochemical outcomes, as shown in our study.

A strength of this study was the NEPSA development process, which surveyed the needs of CRC patients in phase I of our study. NEPSA can be used on all mobile operating systems (Android and iOS), which reduces accessibility or selection bias. Match-group comparison was designed at the beginning of intervention to reduce confounding variables in the quasi-experiment [48]. A professional multidisciplinary team comprising oncologists, nutritionists, oncology nurses, dietitians, and app developers collaborated on this project. Ethical and moral guidelines were strictly applied in this study to reduce researcher bias during implementation. In Thai hospitals, old-fashioned nutritional tools like paper-based leaflets are commonly used. During the COVID-19 pandemics, social distancing policies were promoted throughout the outbreak. NEPSA has the potential to work a responsive, online tool that can facilitate access to nutrition information and support policies. Conversely, a limitation was the small sample size, which was associated with the inclusion criteria for malnutrition in CRC patients (BMI < 20 kg/m^2^) because most of CRC patients are obese. Nonetheless, our study’s sample size was higher than previous studies [24,48]. Our enrollment period lasted over 7 months: this was associated to our study design with group-matching manipulate limitation. Furthermore, this study was a quasi-experiment that cannot control for confounding factors, such as participants’ free-living lifestyles.

For further investigation, we suggest recruiting subjects by sarcopenia risk or percentage of weight loss instead of BMI because finding such participants with CRC will be less difficult. The rehabilitation program, quality of life, and psychological well-being could also be considered.

## 5. Conclusions

Based on our study findings, we can conclude that NEPSA improves nutritional outcomes more efficiently than standard hospital leaflets, as indicated by increases in body weight, BMI, hemoglobin, hematocrit, calorie intake, and protein intake. In addition, NEPSA reduced inflammation by decreasing ESR levels and increasing albumin levels, which was recognized to confer an improvement in nutrition status after three visits. Promoting nutrition literacy in cancer patients helps reduce malnutrition by improving anthropometric and biochemical outcomes in CRC patients. It may also serve as an alternative nutrition educational tool based on mHealth to support Thailand’s digital platform at the national level.

## Figures and Tables

**Table 1:** Baseline characteristics (n = 28).

Demographic Characteristics		Study Group; Number (%)		
		Intervention Group (n = 13)	Comparison Group (n = 15)	*p*-Value
Gender	Male	6 (46.15)	8 (53.33)	1.000
	Female	7 (53.85)	7 (46.67)	
Age (years)	Mean ± SD	63.77 ± 2.26	61.07 ± 2.12	0.877
	41–50	1 (7.69)	2 (13.33)	
	51–60	5 (38.46)	5 (33.33)	
	More than 60	7 (53.85)	8 (53.34)	
Marital status	Single	0 (0)	2 (13.33)	0.484
	Married	13 (100.00)	13 (86.67)	
Education level	Lower than a bachelor’s degree	4 (30.77)	8 (53.33)	0.276
	Bachelor’s degree	9 (69.23)	7 (46.67)	
Monthly income (Bath)	10,000–20,000	4 (30.77)	7 (46.67)	0.355
	20,001–30,000	8 (61.54)	8 (53.33)	
	>30,000	1 (7.69)	0 (0)	
Residences in Thailand	Bangkok and vicinity	10 (76.92)	11 (73.33)	0.100
	Central	1 (7.69)	4 (26.67)	
	Northeastern	2 (15.38)	0 (0)	
Cancer staging	Stage 3	2 (15.38)	3 (20.00)	1.000
	Stage 4	11 (84.62)	12 (80.00)	
Chemotherapy formula	FOLFOX	7 (53.85)	6 (40.00)	0.705
	CAPOX	6 (46.15)	9 (60.00)	

Data are presented in numbers (percentage). There are two chemotherapy regimens: FOLFOX, which consists of oxaliplatin, folinic acid, and fluorouracil. CAPOX consists of oxaliplatin and capecitabine. Significance is indicated by *p*-value < 0.05.

**Table 2:** Comparison of anthropometric parameters (n = 28).

Anthropometric Parameters		Study Group; (Mean ± SD)		
		Intervention Group (n = 13)	Comparison Group (n = 15)	*p*-Value
Body weight (BW) (kg)	Baseline	*48.22* ± *2.12*	*48.06 ± 2.03*	*0.645*
	Visit2	*49.82 ± 2.27* *	*48.6 ± 1.89*	*0.549*
	Visit3	*51.17 ± 2.30* *^,^**	*49.23 ± 11.90*	*0.461*
	BW change (kg)	2.95 ± 0.41	1.17 ± 0.64	0.036
	% BW change	6.13 ± 0.73	2.74 ± 1.28	0.032
BMI (kg/m^2^)	Baseline	*18.26* ± *0.50*	*18.27* ± *0.50*	*0.908*
	Visit2	*18.87 ± 0.52* *	*18.50 ± 0.45*	*0.489*
	Visit3	19.37 ± 0.55 *^,^**	18.67 ± 0.48	0.341
	BMI change (kg/m^2^)	1.11 ± 0.14	0.4 ± 0.21	0.013
	% BMI change	6.10 ± 0.75	2.38 ± 1.21	0.018
Fat %	Baseline	15.43 ± 2.38	16.77 ± 2.21	0.684
	Visit2	17.32 ± 2.28 *	16.61 ± 2.31	0.832
	Visit3	18.01 ± 2.34 *	17.55 ± 2.33	0.892
	% Fat change	6.40 ± 6.36	5.24 ± 3.54	0.062
Fat mass (kg)	Baseline	7.41 ± 1.14	7.8 ± 0.97	0.798
	Visit2	8.6 ± 1.17 *	7.89 ± 1.06	0.655
	Visit3	9.31 ± 1.11 *^,^**	8.51 ± 1.10	0.617
	Fat mass change (kg)	1.89 ± 0.37	0.71 ± 0.26	0.189
	% Fat mass change	8.49 ± 2.30	7.90 ± 4.24	0.357
Muscle mass (kg)	Baseline	38.99 ± 2.27	38.11 ± 2.32	0.79
	Visit2	39.45 ± 2.26 *^,^**	38.44 ± 2.11	0.748
	Visit3	40.38 ± 2.42 *^,^**	38.48 ± 2.08	0.555
	Muscle mass change (kg)	1.39 ± 0.34	0.37 ± 0.52	0.125
	% Muscle mass change	3.52 ± 0.85	1.57 ± 1.30	0.237

Data are expressed by means ± SEM. Italics indicate non-parametric statistical tests. * *p* < 0.05 indicates significant difference within-group compared to baseline. ** *p* < 0.05 indicates significant difference within-group compared to Visit2.

**Table 3:** Comparison of biochemical parameters (n = 28).

Biochemical Parameters		Study Group; (Mean ± SD)		
		Intervention Group (n = 13)	Comparison Group (n = 15)	*p*-Value
Hemoglobin (Hb) (g/dL): Normal range 13.5–17.5 g/dL	Baseline	10.59 ± 0.51	10.61 ± 0.35	0.981
	Visit2	11.15 ± 0.53 *	10.94 ± 0.29	0.726
	Visit3	11.44 ± 0.55 *	10.67 ± 0.41	0.269
	Change Hb	*0.85 ± 0.26*	*0.27 ± 0.28*	*0.070*
Hematocrit (Hct) (%): Normal range 40–52%	Baseline	32.56 ± 1.55	32.67 ± 1.08	0.952
	Visit2	34.11 ± 1.60 *	33.31 ± 0.75	0.659
	Visit3	35.06 ± 1.57 *	32.81 ± 1.47	0.304
	Change Hct	*2.50 ± 0.77*	*0.13 ± 0.88*	*0.090*
Total protein (TP) (g/dL): Normal range 6.6–8.3 g/dL	Baseline	7.12 ± 0.28	7.52 ± 0.22	0.263
	Visit2	*7.61 ± 0.16*	*7.27 ± 0.24*	*0.695*
	Visit3	*7.69 ± 0.22* *	*7.42 ± 0.26*	*0.695*
	Change TP	0.57 ± 0.17	−0.10 ± 0.15	0.008
Globulin (g/dL): Normal range 2.5–3.5 g/dL	Baseline	3.67 ± 0.24	3.82 ± 0.17	0.597
	Visit2	3.80 ± 0.19	3.61 ± 0.18	0.488
	Visit3	3.87 ± 0.26	3.57 ± 0.18	0.353
	Change Globulin	0.20 ± 0.09	−0.25 ± 0.12	0.009
Albumin (g/dL): Normal range 3.5–5.2 g/dL	Baseline	3.41 ± 0.09	3.66 ± 0.16	0.174
	Visit2	3.78 ± 0.08 *	3.65 ± 0.16	0.490
	Visit3	3.85 ± 0.06 *	3.85 ± 0.17	0.999
	Change Albumin	0.43 ± 0.08	0.18 ± 0.07	0.026
ESR (mm/hour): Normal range 0–15 mg/dL	Baseline	65.61 ± 10.27	60.73 ± 10.37	0.742
	Visit2	52.15 ± 10.45	45.80 ± 8.90	0.645
	Visit3	47.61 ± 8.29 *	55.00 ± 10.48	0.594
	Change ESR	−18.00 ± 6.17	−5.13 ± 8.19	0.254

Data are expressed by mean ± SEM. Italics indicate non-parametric statistical tests. * *p* < 0.05 indicates significant difference within-group compared to baseline.

**Table 4:** Comparison of calorie and protein intake (n = 28).

Dietary Parameters		Study Group; (Mean ± SD)		
	Intervention Visit	Intervention Group (n = 13)	Comparison Group (n = 15)	*p*-Value
Total Calories (kcal/day)	Baseline	1344.79 ± 72.55	1239.15 ± 66.71	0.293
	Visit2	1603.35 ± 71.53 *	1491.84 ± 79.24 *	0.312
	Visit3	1797.06 ± 84.99 *^,^**	1486.37 ± 100.14	0.028
	Calories change (Visit3-Baseline)	452.27 ± 56.87	247.22 ± 106.91	0.105
	% Calorie change (Visit3—Baseline)	35.17 ± 5.27	23.62 ± 9.22	0.288
Protein intake (g/day)	Baseline	48.44 ± 3.86	46.72 ± 3.13	0.729
	Visit2	*69.16 ± 4.17* *	*56.13 ± 3.73*	*0.018*
	Visit3	80.68 ± 5.55 *^,^**	61.14 ± 3.82 *	0.006
	A gram of protein change (Visit3—Baseline)	32.24 ± 4.68	14.42 ± 4.09	0.008
	% Protein change (Visit3—Baseline)	74.35 ± 13.52	36.46 ± 9.44	0.027

Data are expressed by means ± SEM. Italics indicate non-parametric statistic tests. * *p* < 0.05 indicates significant difference within-group compared to baseline. ** *p* < 0.05 indicates significant difference within-group compared to Visit2.

## Data Availability

Data is contained within the article.
